# Brain metabolites are associated with sleep architecture and cognitive functioning in older adults

**DOI:** 10.1093/braincomms/fcae245

**Published:** 2024-07-19

**Authors:** Christina Mueller, Rodolphe Nenert, Corina Catiul, Jennifer Pilkington, Jerzy P Szaflarski, Amy W Amara

**Affiliations:** Department of Neurology, University of Alabama at Birmingham, Birmingham, AL 35233, USA; Department of Neurology, University of Alabama at Birmingham, Birmingham, AL 35233, USA; Department of Neurology, University of Alabama at Birmingham, Birmingham, AL 35233, USA; Department of Neurology, University of Alabama at Birmingham, Birmingham, AL 35233, USA; Department of Neurology, University of Alabama at Birmingham, Birmingham, AL 35233, USA; Department of Neurology, University of Alabama at Birmingham, Birmingham, AL 35233, USA; Department of Neurology, University of Colorado Anschutz Medical Campus, Aurora, CO 80045, USA

**Keywords:** brain temperature, cognition, older adults, brain metabolites, magnetic resonance spectroscopy

## Abstract

Sleep deficits are a possible risk factor for development of cognitive decline and dementia in older age. Research suggests that neuroinflammation may be a link between the two. This observational, cross-sectional study evaluated relationships between sleep architecture, neuroinflammation and cognitive functioning in healthy older adults. Twenty-two adults aged ≥60 years underwent whole-brain magnetic resonance spectroscopic imaging (*in vivo* method of visualizing increased brain temperatures as a proxy for neuroinflammation), supervised laboratory-based polysomnography, and comprehensive neurocognitive testing. Multiple regressions were used to assess relationships between magnetic resonance spectroscopic imaging-derived brain temperature and metabolites related to inflammation (choline; *myo*-inositol; *N*-acetylaspartate), sleep efficiency, time and % N3 sleep and cognitive performance. Choline, *myo*-inositol and *N*-acetylaspartate were associated with sleep efficiency and cognitive performance. Higher choline and *myo*-inositol in the bilateral frontal lobes were associated with slower processing speed and lower sleep efficiency. Higher choline and *myo*-inositol in bilateral frontoparietal regions were associated with better cognitive performance. Higher *N*-acetylaspartate around the temporoparietal junction and adjacent white matter was associated with better visuospatial function. Brain temperature was not related to cognitive or sleep outcomes. Our findings are consistent with the limited literature regarding neuroinflammation and its relationships with sleep and cognition in older age, which has implicated ageing microglia and astrocytes in circadian dysregulation, impaired glymphatic clearance and increased blood–brain barrier integrity, with downstream effects of neurodegeneration and cognitive decline. Inflammatory processes remain difficult to measure in the clinical setting, but magnetic resonance spectroscopic imaging may serve as a marker of the relationship between neuroinflammation, sleep and cognitive decline in older adults.

## Teaser

Mueller *et al*. report associations between brain temperature- and metabolite-related sleep and cognition in healthy older adults. Neuroinflammation is emerging as an important contributor to age-related cognitive decline and sleep abnormalities. This study shows that 3D magnetic resonance spectroscopy may be a sensitive biomarker for assessing these relationships.

## Graphical Abstract

**Graphical Abstract:**
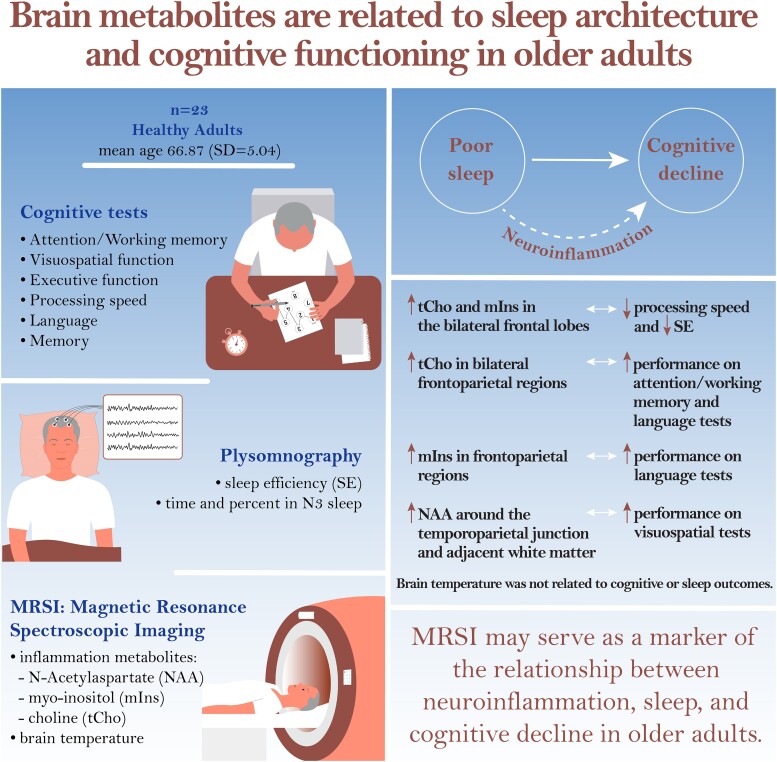

## Introduction

Cognitive dysfunction affects a large proportion of the older population. Approximately 47 million individuals worldwide were living with dementia in 2015, and this number is expected to exceed 135 million by 2050.^1^ Dementia is a leading cause of death worldwide,^2^ reduces quality of life,^3,4^ increases caregiver burden^5^ and risk of institutionalization^6^ and has significant negative socioeconomic impacts.^7,8^ Cognitive decline is also experienced by many older adults who do not go on to develop dementia.

Advancing age and cardiovascular disease are risk factors for cognitive dysfunction.^9,10^ Increasing evidence also points to sleep deprivation as a significant risk factor for cognitive decline and dementia in older age.^11,12^ The American Association of Sleep Medicine recommends at least 7 h of sleep per night for adults aged 18 years and over,^13^ with between 6% and 30% of individuals estimated to sleep for inadequate amounts of time.^14^ In particular, slow wave sleep [non-rapid eye movement (NREM) stage 3 (N3)] is important for memory consolidation during sleep and influences cognitive function in healthy adults and adults with mild cognitive impairment.^15,16^ However, some studies have found that both shortened and excessively long sleep durations could be detrimental to cognitive functioning in older adults.^17-19^ It is also important to note that the relationship between sleep disturbances and dementia is still ambiguous, and not all studies have found evidence for an association between the two.^20^

Neuroinflammation plays an important role in the pathogenesis of cognitive dysfunction and may serve as a link between sleep deprivation and cognitive decline.^21-23^ Importantly, evidence of neuroinflammation exists in the ageing brain prior to progression to dementia and therefore may be detectable as a prodromal or predictive feature of cognitive decline.^24,25^ Despite significant efforts, currently no pharmacological agents are available to reverse cognitive dysfunction in the elderly, although several recently developed drugs such as lecanemab^26^ and donanemab^27^ show promise for slowing cognitive decline in Alzheimer’s disease. Modification of risk factors such as sleep deprivation may modulate neuroinflammation, thus reducing long-term susceptibility to cognitive dysfunction and dementia.^9,28^ However, more work is needed to understand associations between sleep and neuroinflammation and how these relate to cognitive decline in older adults.

Measuring neuroinflammation in living humans poses significant challenges, and much of what is known has been acquired through post-mortem brain samples or invasive procedures such as brain biopsy or lumbar puncture.^24,25^ Positron emission tomography radioligands for the translocator protein have been designed to detect neuroinflammation, but these studies are often cost prohibitive and not readily available.^24,29^ Magnetic resonance spectroscopic imaging (MRSI) has been proposed as a potential non-invasive alternative to measuring neuroinflammation.^30,31^ With this technique, brain temperature can be measured as a proxy for neuroinflammation,^32^ based on the observation that metabolic changes during inflammation cause local increases in temperature. MRSI-based brain thermometry is reliable^33,34^ and valid^35,36^ and can detect low-grade neuroinflammation.^32,37^ In addition to brain temperature, MRSI quantifies several metabolites associated with neuroinflammatory processes. The choline (tCho) metabolite peak, which consists of phosphorylcholine and glycerophosphorylcholine compounds, is a component of the cell membrane, and elevated tCho levels in the brain indicate increased cellular membrane turnover, which can be seen under inflammatory conditions.^38^  *Myo*-inositol (mIns) is a glial marker, and increased mIns indicates glial proliferation and activation in inflamed brain tissue.^38^  *N*-Acetylaspartate (NAA) is a neuronal marker.^39^ This metabolite is not directly linked to the neuroinflammatory process, but can be decreased due to neuronal loss that often accompanies neuroinflammation. Thus, in the current study, we measured these metabolites and brain temperature as putative markers of neuroinflammation.

MRSI has the potential to detect pathogenic brain changes involving neuroinflammation in older adults early in the development of cognitive decline. Prior studies of individuals who go on to develop dementia have shown that pathological changes such as metabolic changes, amyloid-β deposition and brain atrophy are detectable years or even decades before diagnosis,^40,41^ which puts early detection and intervention within reach. To better understand the role of sleep and neuroinflammation and their interplay in cognitive decline, the current study aimed to explore relationships between sleep architecture, brain temperature and metabolites and cognitive performance in older adults. We hypothesized that higher brain temperature, tCho and mIns and lower NAA would be associated with worse cognitive performance and worse objective sleep outcomes [measured with polysomnography (PSG)]. Investigating the relationship between neuroinflammation and cognitive function may inform the development of more definitive studies using MRSI as a cross-sectional and longitudinal biomarker of neuroinflammation in older adults at risk of cognitive impairment or dementia.

## Materials and methods

### Design

In this single-group, cross-sectional and prospective observational study, we tested the relationship between brain temperature, metabolites (tCho, mIns and NAA), sleep and cognitive outcomes. Cognitive measures included *Z*-scores in domains of processing speed, attention/working memory, language, memory, visuospatial function and executive function. Sleep outcomes were sleep efficiency (SE), N3 time and N3%.

### Participants

Participants aged at least 60 years were recruited via flyers, online advertisements and word-of-mouth. A score of at least 18 out of 30 on the Montreal Cognitive Assessment (MoCA) was also an inclusion criterion. The following exclusion criteria applied: any neurological disease, including Alzheimer’s disease, Parkinson’s disease, stroke within 1 year of participating and multiple sclerosis; uncontrolled cardiovascular or pulmonary disease, including untreated hypertension, congestive heart failure, coronary artery disease, arrhythmia and chronic obstructive pulmonary disease; untreated sleep apnoea or another sleep disorder including rapid eye movement (REM) sleep without atonia; shift work; chronic anti-inflammatory medication use; medication adjustments within 1 month of participating; MRI contraindications (e.g. ferromagnetic implants and severe claustrophobia); and other medical conditions that could have prevented participants from performing study procedures.

### Study procedures

The study protocol was approved by the Institutional Review Board of the University of Alabama at Birmingham (UAB). Participants provided written informed consent, and all procedures were performed according to the Declaration of Helsinki. Participation involved a screening visit followed by the study visit.

#### Cognitive assessment

Participants underwent a comprehensive cognitive evaluation with at least two tests in each of the following domains: (i) Memory domain: (a) the 10/36 Spatial Recall Test^42^ and (b) Hopkins Verbal Learning Test—Revised (HVLT; PAR, Inc., Lutz, FL, USA); (ii) Executive function domain: (a) inhibition and (b) inhibition/switching subtests of the Stroop Colour and Word Test (SCWT)^43^ of the Delis–Kaplan Executive Function System (D-KEFS; Pearson Assessments, London, UK)^44^; and (c) Trail Making Test (TMT) Trails B-A^45,46^; (iii) Language: (a) Boston Naming Test (second edition),^47^ (b) Controlled Oral Word Association Test (COWAT) and (c) Animal Naming Test^48,49^; (iv) Attention/working memory domain: (a) Digit span (forward and backward) and (b) letter–number sequencing subtests of the Wechsler Adult Intelligence Scale (third edition) (WAIS-III)^50^; (v) visuospatial function domain: (a) Benton judgment of line orientation (JLo; Form V; PAR, Inc., Lutz, FL, USA) and (b) the Hooper Visual Organization Test (VOT; WPS, Torrance, CA, USA); and (vi) processing speed domain: (a) Stroop colour naming, (b) Stroop word naming and (c) Trails A. Raw scores from each test were converted to normalized *Z*-scores based on normative values. Test-specific norms were used for the SCWT of the D-KEFS,^44^ HVLT and WAIS-III,^50^ which adjust scores for participant age. JLo scores were adjusted for sex, and VOT scores were adjusted for age and sex. Age-, gender- and education-adjusted norms^49^ were used for the 10/36 Spatial Recall Test. The COWAT, Animal Naming Test and TMT were adjusted for age, sex, education and race/ethnitcity.^49^ Normalization was conducted according to the test manual or published population norms; thus, by test design, *Z*-scores differ with regard to which factors (age, sex, education and race/ethnicity) were taken into account. In all cases, higher *Z*-scores indicate better functioning. In a final step, *Z*-scores for individual tests within each domain were averaged to obtain domain scores. HVLT memory score (sum of HVLT total recall and discrimination index) was also calculated.

#### Polysomnography

On the day following the cognitive assessment, overnight video PSG was performed at the UAB Sleep and Wake Disorders Center. Time between the cognitive assessments and PSG was kept consistent across participants. Electroencephalography was performed with leads F3, F4, C3, C4, O1 and O2 referenced to contralateral mastoid. Electromyogram was performed at the submental and bilateral anterior tibialis and extensor digitorum communis and airflow monitoring with thermocouple and nasal pressure. We also collected electrooculogram and respiratory effort using polyvinylidene fluoride belts at the chest and abdomen and pulse oximetry. The examinations were scored by a certified sleep technician and a Sleep Medicine board-certified physician (A.W.A.). SE (percentage of time spent asleep in relation to total time spent in bed, with normal levels being 85% or higher), N3 time (number of minutes of N3 sleep during the PSG) and N3% (minutes of N3 divided by total minutes of sleep) were quantified from the PSG. N3 is the deepest stage of NREM sleep and characterized by regular heart rate and respiration and a predominance of delta brain waves. N3 sleep predominates during the first third of the night and decreases with age.

#### Neuroimaging

On the morning following the PSG, neuroimaging was performed on a 3-T Siemens Prisma Magnetom (Siemens Healthineers, Erlangen, Germany) with 64-channel head and neck coil. A magnetization-prepared rapid gradient echo was acquired with the following parameters: repetition time (TR) = 2400 ms, echo time (TE) = 2.22 ms, flip angle = 8°, slices = 208, thickness = 0.8 mm, matrix = 256 × 256 and final voxel resolution = 0.8 mm^3^ isotropic. A fast 3D echoplanar spectroscopic imaging (EPSI) sequence was acquired with TR_1_ = 1550 ms, TR_2_ = 511 ms, TE = 17.6 ms, inversion time for lipid signal suppression = 198 ms, GRAPPA acceleration factor = 1.3, flip angle = 71°, field-of-view = 280 × 280 × 180 mm and matrix = 50 × 50 × 18. A saturation band was used to suppress signal from the eyes and sinus cavities. Iterative shimming and off-resonance frequency adjustment were performed prior to acquisition. The EPSI sequence acquires metabolite data and a water reference image using two TRs. Water suppression was applied during acquisition of the metabolite spectra, while no suppression was applied during acquisition of the water reference image.

### Image processing

The metabolite imaging and data analysis system (MIDAS) was used to process the EPSI data.^51^ Raw data were reconstructed, corrected for b_0_ inhomogeneities and smoothed. The *REFDAT* module in MIDAS was used to create a B0 map from the water reference image, and correction for spatially dependent B0 shifts was implemented in the *FDFT* module using the B0 maps as input. Spatial smoothing of the metabolite maps was performed in the *LITE* module using spatial convolution and a Gaussian filter of 7 mm in plane and 5 mm through plane. Spectral fitting was performed with the *FITT2* module. Temperature was calculated based on the chemical shift of the unsuppressed water peak relative to the methyl creatine (CRE) peak, which resonates at 3.03 ppm, as shown in the following equation:


$$T_{\mathrm{CRE}}=-102.61\times \Delta_{H2O\text{-}\mathrm{CRE}}+206.1^{\circ }C$$

The anatomical images were segmented, and temperature calculations were corrected for grey and white matter content.^52^ The metabolite maps were expressed as ratios over total creatine (tCr) using the *SINorm* module in MIDAS. Maps were then interpolated to 4.375 × 4.375 × 5.625 mm resolution, non-linearly warped to Montreal Neurological Institute 2 mm space and exported from MIDAS. Voxels with linewidths outside of the 2.0–12.0 Hz range or cerebrospinal fluid content of more than 30% were excluded. Smoothing was performed with AFNI’s (version 20.1.18) *3dMedianFilter* using a two-voxel radius and additionally with an 8-mm Gaussian kernel in SPM12 (https://www.fil.ion.ucl.ac.uk/) for MATLAB (v. R2021a, MathWorks, Natick, MA, USA). Voxels with temperatures outside the 35.0–44.0°C range were excluded as an additional quality control measure, as these temperatures are physiologically implausible. As exported, the temperature maps are scaled so that a raw value of 3 corresponds to 35°C (95°F). The voxelwise addition of 32 results in maps reflecting the corresponding value in °C.

### Statistical analyses

Skewness, kurtosis and frequency plots were assessed for all outcomes to test data normality. Pearson’s correlation coefficients were obtained for normally distributed data, and Spearman’s *ρ* was used in the case of non-normal data.

Correlation coefficients were obtained to assess the bivariate relationships between the sleep variables (N3 time, N3% and SE) and the cognitive outcomes (domain scores and HVLT-based memory score). Correlation coefficients were deemed significant at *P* < 0.05 (uncorrected).

For region-of-interest (ROI) analyses, masks of the left and right dorsolateral prefrontal cortex (dlPFC) (Brodmann areas 9 and 46), left and right hippocampus and left and right thalamus were created from the TT_Daemon atlas in AFNI. Mean temperature and metabolite ratios (tCho/tCr, mIns/tCr and NAA/tCr) were extracted from the ROIs with the *3dROIstats* function in AFNI. Based on suspected relationships between brain regions and clinical outcomes, Pearson’s correlation coefficients were obtained in IBM SPSS Statistics for Macintosh, version 28.0 (IBM Corp., Armonk, NY, USA), between the following outcomes: brain temperature and metabolites in the left and right dlPFC and the executive functioning and processing speed domain scores; temperature and metabolites in the left and right hippocampus and the HVLT-based memory score and the memory domain score; and temperature and metabolites in the left and right dlPFC, hippocampus and thalamus and the sleep outcomes (N3 time, N3% and SE). The brain regions were selected based on prior literature showing involvement of the dlPFC in executive functioning and processing speed,^53,54^ the hippocampus and memory^55,56^ and the prefrontal cortex,^57,58^ hippocampus^15,59^ and thalamus^60-62^ in sleep. Correlations were deemed significant at *P* < 0.0101 (corrected for 78 tests using a false discovery rate of *α*=0.05).^63^

For exploratory whole-brain analyses, multiple linear regressions were performed using AFNI’s *3dttest++* function. Separate regressions were conducted for each cognitive domain score, the HVLT-based memory score and each sleep outcome. Participant age and sex were added as nuisance regressors to account for statistical variance associated with these factors and to increase statistical power to detect associations with variables of interest (sleep and cognitive function). A whole-brain mask was used to exclude non-brain voxels from the analyses. The *3dFWHM* function in AFNI was used on the regression model residuals to calculate estimates of the spatial noise in the temperature and metabolite maps. The noise estimates were then used with AFNI’s *3dClustSim* function to determine thresholds for significant clusters. We used nearest-neighbour 1 clustering and bi-sided thresholding with a voxel-level threshold of *P* < 0.01 and cluster-level threshold of *α*=0.05. Finally, AFNI’s *3dROIstats* function was used to extract mean values from significant clusters for each participant, and these were plotted against behavioural (cognition and sleep) variables to visualize significant relationships. Example code used to run *3dttest++*, *3dFWHM*, *3dClustSim* and *3dROIstats* is included in the supplementary materials.

## Results

### Participant characteristics

A flowchart of included and excluded participants is included in the supplementary materials. Forty-one potential participants were prescreened for initial inclusion criteria, and 35 of them were scheduled for a screening visit. One person was lost to follow-up and 34 attended the screening visit. Nine participants were excluded during screening for the following reasons: MRI contraindications (*n* = 3), possible sleep apnoea (desaturation index >5 events/hour as measured by nocturnal pulse oximetry; *n* = 4), diagnosis of REM Behaviour Disorder (*n* = 1) and planned surgery that would have necessitated medication changes during study participation (*n* = 1). Twenty-five participants qualified and attended the study visit. Of them, one data set was excluded for ceasing to maintain eligibility (sleep apnoea detected during PSG), and two did not provide usable imaging data, meaning that 22 complete datasets were included in the final analyses. There were no missing data on the cognitive testing. One participant did not undergo PSG, so their data were not included in the analysis of sleep data. The mean age of participants was 66.91 years (SD = 5.15). Nine participants were men and 13 were women. Participant characteristics and sleep and cognitive scores are summarized in Table 1.

**Table 1:** Participant characteristics, sleep outcomes and cognitive domain scores

	Mean	SD
Age	66.91	5.15
Sex	9 men (41%); 13 women (59%)	
MoCA	26.32	2.68
HVLT-based memory score	34.57	5.56
Executive functioning domain	0.76	0.52
Attention/working memory domain	0.62	0.60
Memory domain	−0.16	0.62
Language domain	0.58	0.62
Visuospatial domain	0.67	0.94
Processing speed domain	0.50	0.66
SE (%)	78.28	11.35
N3 time (min)	71.81	31.26
N3%	18.98	7.84

Domain scores are *Z*-scores.

### Data normality

Full results from normality testing are included in the supplementary materials. Normality testing showed that the behavioural variables were normally distributed. For neuroimaging outcomes, testing indicated deviations from normality for the following: tCho in the left and right dlPFC, mIns in the left hippocampus and right dlPFC and NAA in the left dlPFC. For those brain regions and metabolites, Spearman’s *ρ* (for non-normal data) was obtained and reported instead of Pearson’s *r*.

### Relationships between sleep and cognition

There were no significant correlations between the sleep variables (SE, N3 time and N3%) and the cognitive domain scores or HVLT-based memory score (all *P* > 0.05).

### Main imaging results (*a priori* hypotheses)

Main imaging results are summarized in Table 2. Integrated spectra from regions of interest in a representative participant are included in the supplementary materials. There were no significant correlations between brain temperature or metabolites in the dlPFC and the executive functioning or processing speed domains (all *P* > 0.0101). There were no correlations between temperature or metabolites in the hippocampus and the memory outcomes (*P* > 0.0101). There were no significant correlations between brain temperature or metabolites and the sleep outcomes (*P* > 0.0101).

**Table 2:** Correlation coefficients (and associated *P*-values) for associations between brain temperature and metabolites in ROIs and cognitive function and sleep outcomes

		Brain temperature	tCho/tCr	mIns/tCr	NAA/tCr
HVLT-based memory score					
L hippocampus		0.350 (0.110)	−0.248 (0.265)	−0.400^a^ (0.065)	−0.337 (0.125)
R hippocampus		0.063 (0.782)	−0.187 (0.405)	−0.409 (0.059)	−0.440 (0.040)
Executive functioning domain					
L dlPFC		0.288 (0.139)	0.274^a^ (0.217)	−0.081 (0.721)	0.233^a^ (0.296)
R dlPFC		0.184 (0.411)	−0.074^a^ (0.744)	−0.123^a^ (0.587)	−0.092 (0.685)
Memory domain					
L hippocampus		0.029 (0.899)	0.051 (0.822)	−0.018^a^ (0.938)	−0.459 (0.032)
R hippocampus		−0.039 (0.862)	0.007 (0.975)	−0.163 (0.470)	−0.311 (0.159)
Processing speed domain					
L dlPFC		−0.004 (0.986)	−0.219^a^ (0.327)	−0.076 (0.736)	−0.120^a^ (0.595)
R dlPFC		0.130 (0.565)	−0.302^a^ (0.172)	−0.236^a^ (0.291)	−0.101 (0.655)
SE					
L dlPFC		−0.042 (0.856)	0.044^a^ (0.849)	−0.098 (0.673)	−0.110^a^ (0.634)
R dlPFC		−0.206 (0.370)	−0.287^a^ (0.207)	0.068^a^ (0.771)	−0.228 (0.321)
L hippocampus		−0.012 (0.958)	−0.241 (0.292)	−0.271^a^ (0.234)	−0.198 (0.390)
R hippocampus		0.056 (0.810)	−0.259 (0.257)	0.040 (0.863)	−0.046 (0.844)
L thalamus		−0.107 (0.644)	−0.401 (0.072)	−0.211 (0.359)	−0.233 (0.310)
R thalamus		−0.038 (0.870)	−0.298 (0.190)	−0.249 (0.277)	−0.135 (0.559)
N3 time					
L dlPFC		0.178 (0.440)	0.021^a^ (0.929)	−0.126 (0.586)	−0.121^a^ (0.602)
R dlPFC		0.152 (0.510)	0.031^a^ (0.893)	0.006^a^ (0.978)	−0.151 (0.514)
L hippocampus		0.151 (0.515)	−0.194 (0.400)	−0.017^a^ (0.942)	−0.221 (0.335)
R hippocampus		0.445 (0.043)	0.145 (0.530)	0.229 (0.318)	−0.129 (0.577)
L thalamus		0.079 (0.732)	−0.320 (0.158)	0.238 (0.298)	−0.008 (0.972)
R thalamus		0.143 (0.537)	−0.327 (0.148)	0.222 (0.334)	−0.043 (0.852)
N3%					
L dlPFC		0.184 (0.424)	0.100^a^ (0.666)	−0.055 (0.813)	0.058^a^ (0.801)
R dlPFC		0.233 (0.310)	0.127^a^ (0.582)	0.082^a^ (0.724)	−0.065 (0.781)
L hippocampus		0.164 (0.477)	−0.139 (0.549)	0.169^a^ (0.464)	−0.199 (0.388)
R hippocampus		0.488 (0.025)	0.252 (0.271)	0.288 (0.205)	−0.102 (0.660)
L thalamus		0.106 (0.647)	−0.263 (0.249)	0.358 (0.111)	0.047 (0.840)
R thalamus		0.163 (0.480)	−0.291 (0.201)	0.354 (0.115)	−0.007 (0.975)

^a^Spearman’s *ρ* for non-normally distributed data. Correlation coefficients are Pearson’s *r* unless otherwise indicated. Domain scores are *Z*-scores.

L, left; R, right.

### Whole-brain imaging results (exploratory)

**Figure 1:**
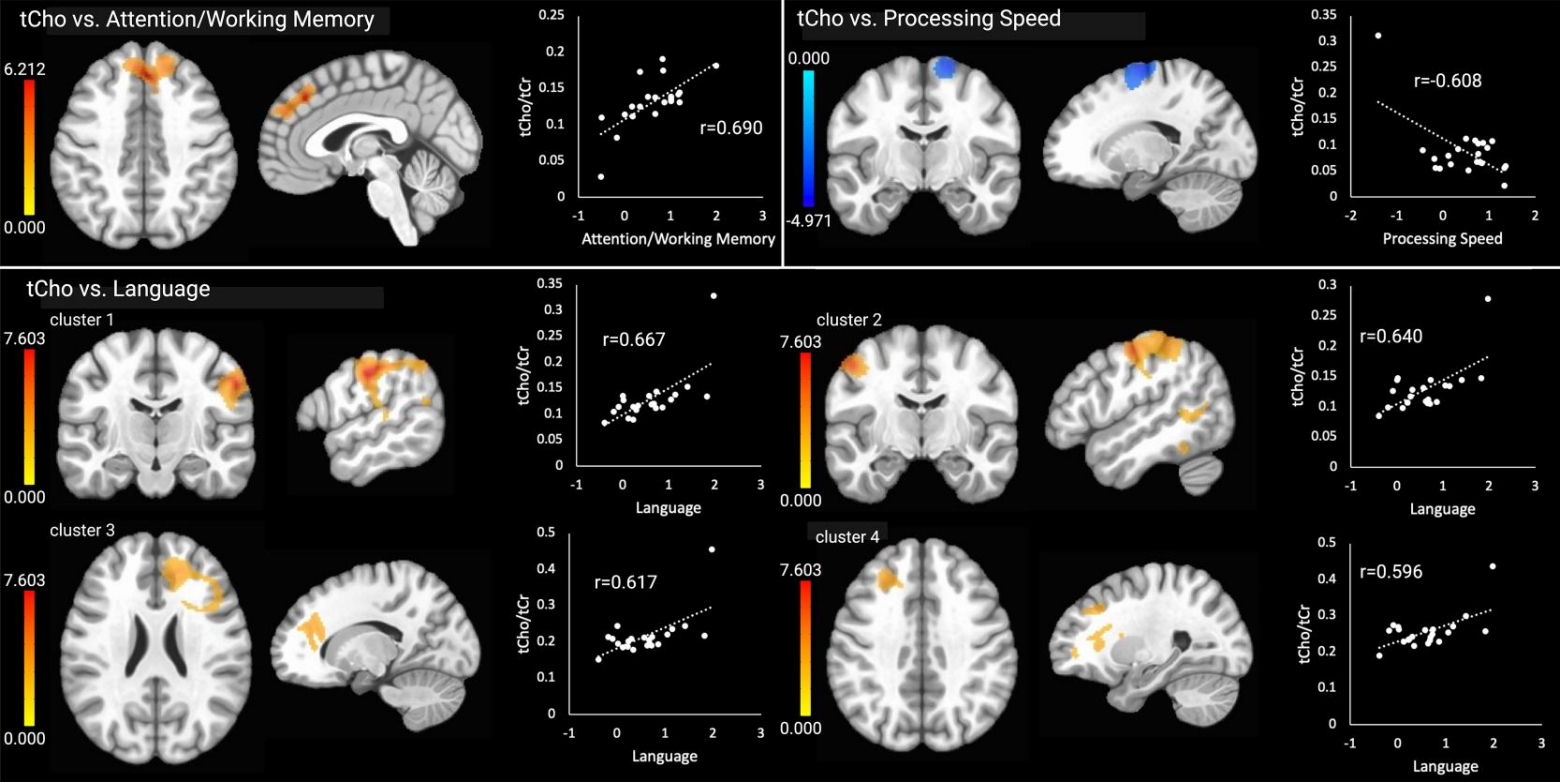
**Significant relationships between tCho and the cognitive outcomes.** Brain images are shown in neurological convention (left = left). Colour bars indicate *t*-values from multiple regressions. *r*-values represent effect sizes (Pearson’s coefficients) for relationships between mean cluster signal and cognitive scores. The *x*-axis shows cognitive domain *Z*-scores.

Clusters with significant correlations are shown in Table 3. There were no significant relationships between brain temperature and the cognitive outcomes. Higher tCho (i.e. increased cellular turnover) in the frontal lobe (frontal pole, superior frontal gyrus and paracingulate gyrus) was related to higher (better) attention/working memory scores, and higher tCho in four clusters was associated with higher language scores. These included the right parietal and occipital lobes including the temporoparietal junction (cluster 1), left parietal lobe, temporoparietal junction and frontal motor areas (cluster 2), right frontal cortex and white matter (cluster 3) and left frontal cortex and white matter (cluster 4). There were significant associations between higher tCho in frontal motor areas and worse processing speed scores. The tCho results are shown in Fig. 1. There were no clusters with significant relationships between tCho and executive functioning, memory or visuospatial skills.

**Table 3:** Clusters with significant relationships between metabolites and clinical outcomes

	Hem.	Region	MNI coordinates (peak signal)			Cluster size (mm^3^)
tCho						
tCho × attention/WM						
Cluster 1 (+)	bil	Frontal pole, sup. frontal gyrus and paracingulate gyrus	+2	+34	+42	1681
tCho × language						
Cluster 1 (+)	R	Postcentral gyrus, ant. supramarginal gyrus, post. supramarginal gyrus, angular gyrus, sup. lateral occipital gyrus, intracalcarine cortex and occipital fusiform gyrus	+58	−16	+40	4488
Cluster 2 (+)	L	Precentral gyrus, post. sup. temporal gyrus, postcentral gyrus, ant. supramarginal gyrus, post. supramarginal gyrus and angular gyrus	−50	−10	+50	2820
Cluster 3 (+)	R	Frontal pole, middle frontal gyrus, paracingulate gyrus, ant. corona radiata, ant. thalamic radiation and forceps minor	+16	+40	+24	2145
Cluster 4 (+)	L	Frontal pole, middle frontal gyrus, inf. frontal gyrus (pars triangularis), genu of corpus callosum, ant. corona radiata, ant. thalamic radiation, forceps minor and inf. fronto-occipital fasc	−26	+32	+38	1775
tCho × Proc. Speed						
Cluster 1 (−)	R	Sup. frontal gyrus, precentral gyrus and juxtapositional lobule (SMA)	+18	−10	+72	1717
tCho × SE						
Cluster 1 (−)	bil	Sup. frontal gyrus, middle frontal gyrus, precentral gyrus and juxtapositional lobule (SMA)	+12	+2	+56	2859
mIns						
mIns × Language						
Cluster 1 (+)	R	Precentral gyrus, postcentral gyrus, ant. supramarginal gyrus, post. supramarginal gyrus, angular gyrus, sup. lateral occipital cortex, inf. lateral occipital cortex and sup. longitudinal fasc.	+64	−22	+40	4682
Cluster 2 (+)	L	Middle frontal gyrus, precentral gyrus, postcentral gyrus and CST	−62	−22	+48	1544
mIns × Proc. Speed						
Cluster 1 (−)	bil	Sup. frontal gyrus, middle frontal gyrus and juxtapositional lobule (SMA)	+24	−16	+76	5017
mIns × SE						
Cluster 1 (−)	bil	Sup. frontal gyrus, middle frontal gyrus, precentral gyrus and juxtapositional lobule (SMA)	+26	−4	+62	8625
NAA						
NAA × V/s function						
Cluster 1 (+)	L	Post. sup. temporal gyrus, post. middle temporal gyrus, temporo-occipital middle temporal gyrus, temporo-occipital inf. temporal gyrus, temporal occipital fusiform cortex, post. thalamic radiation, sagittal stratum, inf. fronto-occipital fasc. and inf. longitudinal fasc.	−44	−14	−16	1829

Only regions with at least 3% cluster overlap are reported. (+) positive association, (−) negative association.

ant., anterior; bil, bilateral; CST, corticospinal tract; fasc., fasciculus; Hem., hemisphere; L, left; post., posterior; Proc. speed, processing speed; R, right; SE, sleep efficiency; SMA, supplementary motor area; sup., superior; V/s, visuospatial; WM, working memory.

**Figure 2:**
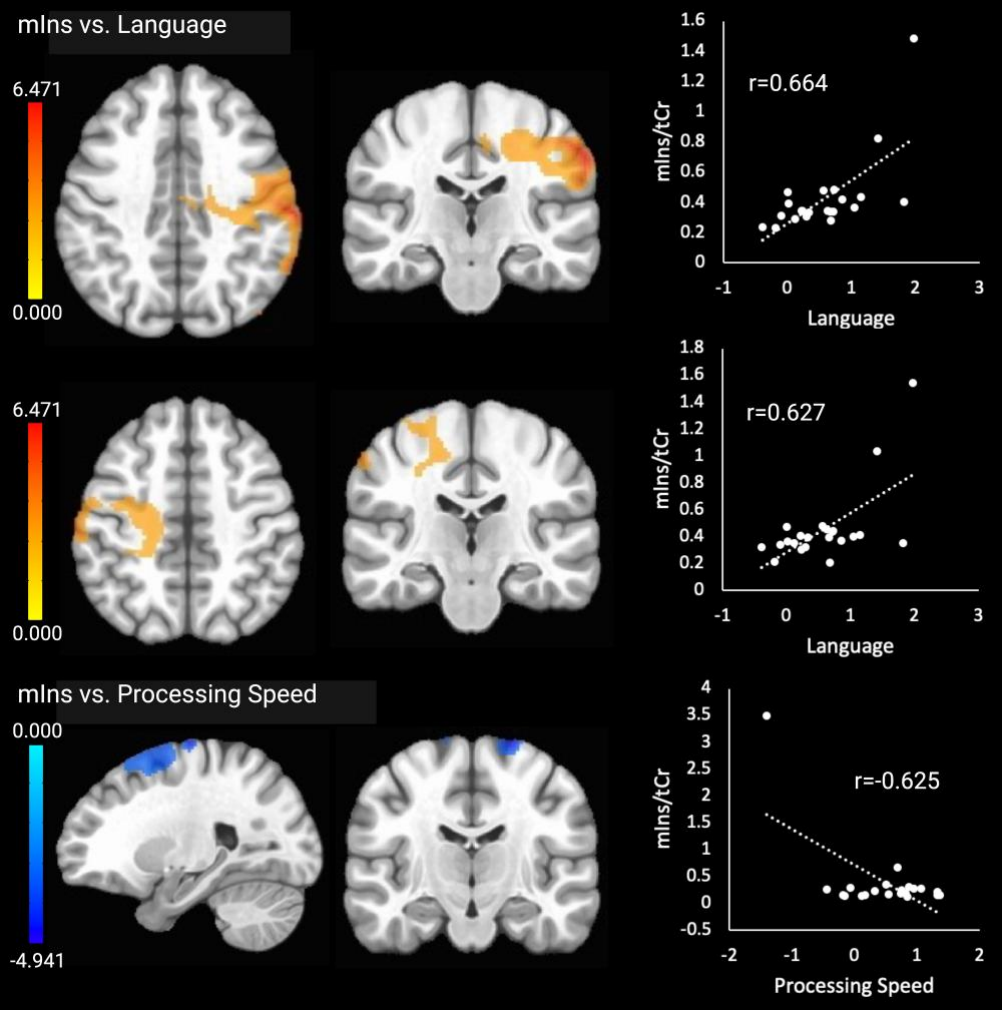
**Significant relationships between mIns and the cognitive outcomes.** Brain images are shown in neurological convention (left = left). Colour bars indicate *t*-values from multiple regressions. *r*-values represent effect sizes (Pearson’s coefficients) for relationships between mean cluster signal and cognitive scores. The *x*-axis shows cognitive domain *Z*-scores.

Higher language scores were associated with higher mIns in two clusters in right frontoparietal areas including motor areas, the temporoparietal junction and occipital regions (cluster 1) and in left middle frontal gyrus, frontal and parietal motor areas and the corticospinal tract (cluster 2). Lower processing speed scores were related to higher mIns in the frontal lobe, including frontal motor areas, a cluster that overlapped with the cluster showing negative correlations between tCho and processing speed. The mIns results are shown in Fig. 2. There were no clusters with significant relationships between mIns and attention/working memory, executive functioning, memory or visuospatial skills.

**Figure 3:**
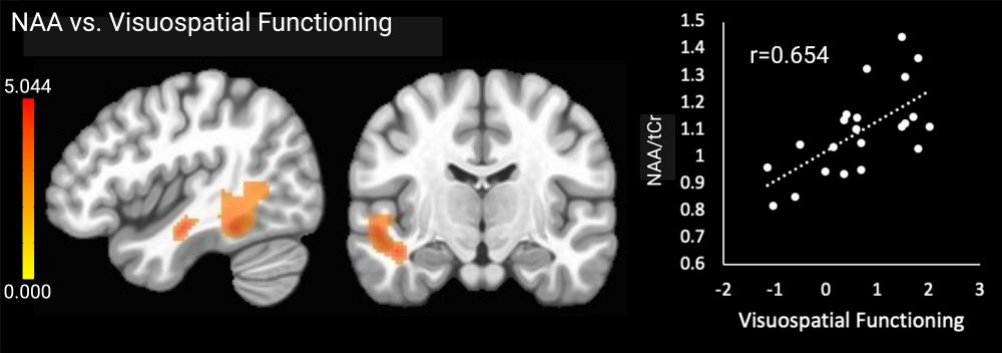
**Significant relationships between NAA and visuospatial functioning.** Brain images are shown in neurological convention (left = left). Colour bars indicate *t*-values from multiple regressions. *r*-values represent effect sizes (Pearson’s coefficients) for relationships between mean cluster signal and cognitive scores. The *x*-axis shows visuospatial domain *Z*-score.

Higher NAA (neuronal health) in one cluster comprising left temporal cortical and white matter regions was associated with higher visuospatial functioning scores. The NAA results are shown in Fig. 3. There were no significant associations with any other cognitive outcomes.

**Figure 4:**
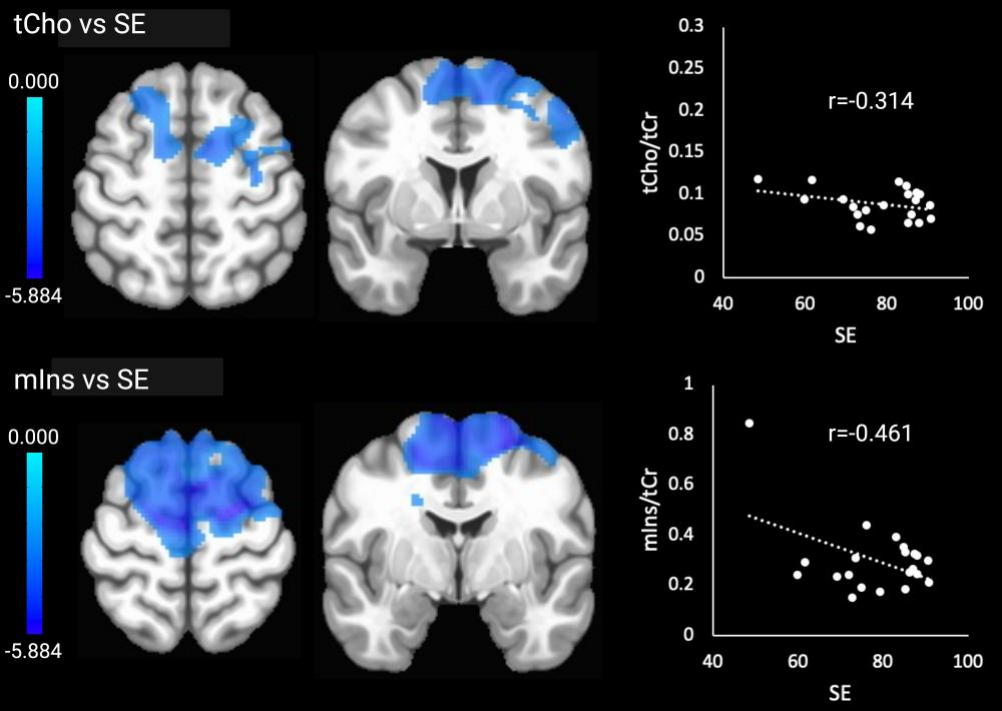
**Significant relationships between tCho and mIns and SE.** Brain images are shown in neurological convention (left = left). Colour bars indicate *t*-values. *r*-values represent effect sizes (Pearson’s coefficients) for relationships between mean cluster signal and SE.

Higher tCho (cellular turnover) in the bilateral frontal lobes was associated with lower SE. Higher mIns (glial activation) in the same regions was also associated with lower SE. tCho and mIns were not associated with N3 time or N3%. Significant associations with the sleep outcomes are shown in Fig. 4. There were no correlations between the sleep outcomes and brain temperature or NAA.

## Discussion

This study aimed to assess associations between sleep architecture, spectroscopic brain temperature and metabolites and cognitive functioning in older adults. There were no significant associations between brain temperature and cognitive performance. We found several associations between brain metabolites (mIns, tCho and NAA) and cognitive performance and SE. Overall, the findings are consistent with the limited literature regarding neuroinflammation and its relationships with sleep and cognition in older age, which has implicated ageing microglia and astrocytes in circadian dysregulation, impaired glymphatic clearance and increased blood–brain barrier integrity, with downstream effects of neurodegeneration and cognitive decline.^64^ The current study was the first to probe associations between whole-brain temperature and sleep and cognitive function in healthy older adults, although we did not observe significant relationships with brain temperature. The identified associations with mIns and NAA point to glial activation and proliferation and neuronal health as predictors of cognitive function in this population. Understanding how lifestyle factors such as sleep are related to healthy brain ageing could lead to more targeted lifestyle modifications in individuals at risk of cognitive decline. Specific findings are discussed in detail below.

The exploratory whole-brain analyses identified overlapping clusters in the bilateral frontal cortex (superior and middle frontal gyrus and supplementary motor area) where higher mIns and higher tCho levels were associated with slower processing speed. mIns is a glial marker and increases in this metabolite are thought to reflect glial activation and proliferation during neuroinflammation, as well as remyelination after injury or neurodegeneration.^38^ mIns increases are consistently reported in Alzheimer’s disease^65,66^ and mild cognitive impairment,^67,68^ with the hippocampus showing the highest increases.^30^ Besides neuroinflammation, mIns increases could also reflect cellular responses to hippocampal atrophy, such as brain plasticity and remyelination. Overlapping associations of mIns (glial marker) and tCho (metabolic marker) suggest that neuroinflammation could be driving the processing speed deficits in the affected areas, but because brain temperature in these areas was not associated with processing speed, this interpretation is also speculative. The findings are consistent with existing literature regarding tCho and mIns abnormalities in older adults, which appear to be a link between the APOε_4_ genetic risk factor and cognitive deficits in Alzheimer’s disease.^69^

Contrary to our hypotheses, we also identified positive associations between tCho levels and the cognitive measures, particularly language skills. Some of the affected regions, such as the middle frontal gyrus and inferior frontal gyrus, are known to be involved in the development of literacy and language processing, which lends credence to our findings. Higher tCho in the superior frontal gyrus and the frontal pole was associated with higher attention and working memory abilities. The superior frontal gyrus has previously been associated with working memory functioning, while the frontal pole is thought to be involved in outcome monitoring.^70-73^ tCho is a component of the cell membrane, and higher levels reflect increased cellular turnover. tCho can be synthesized in the brain or sourced from food; it supports membrane integrity and cholinergic neurotransmission.^74^ Abnormal increases in tCho have been documented in healthy older adults relative to younger adults^75^ and in Alzheimer’s disease compared to healthy older adults.^76^ Neuroinflammation is one mechanism that could lead to abnormally increased tCho, and this was the basis for our hypothesis that higher tCho would be associated with worse memory performance. However, it is important to note that other factors such as dietary tCho intake and ability to synthesize tCho from food also affect tCho levels in the brain. For example, older adults have been shown to exhibit less efficient dietary tCho uptake in the brain than younger adults,^77,78^ and population studies have shown that dietary tCho intake is associated with better cognitive functioning in older adults.^79^ Thus, it is possible that the positive associations between tCho and cognitive functioning we observed could reflect relatively healthier brain functioning and lifestyle factors such as dietary tCho intake, especially in individuals without cognitive impairment, such as our sample. However, this explanation is speculative and the unexpected associations between tCho and better cognitive function should be explored in future studies.

Finally, in line with our hypotheses, we found positive associations between NAA in posterior temporal and occipital regions and visuospatial functioning. The regions included posterior portions of the superior, middle and inferior temporal gyri and the fusiform gyrus. NAA is a marker of neuronal health and density, and the results are consistent with prior studies that have reported NAA decreases throughout the brain in Alzheimer’s disease^65,66,80^ and healthy ageing,^81^ which have been linked to deficits in overall cognitive functioning.^82-84^

We expected that lower SE and lower amounts of N3 sleep would be associated with higher brain temperature, tCho and mIns (indicating inflammation) and worse cognitive performance. In support of our hypotheses, we found significant relationships between higher tCho and mIns in bilateral frontal regions, including the superior and middle frontal gyrus, precentral gyrus and supplementary motor area, and lower SE. The findings suggest that worse sleep in older age is linked to heightened cellular turnover and glial activation, which could reflect the impact of neuroinflammation.

Our findings are consistent with the limited literature regarding neuroinflammation and its impact on sleep in older age. As discussed in a recent review,^85^ astrocytes contribute to the development of neuroinflammation during ageing and thus the development of brain pathology and cognitive decline. In the healthy brain, astrocytes contribute to regulating sleep and circadian rhythms, including glymphatic clearance of neurotoxic substances during sleep and reducing network excitability during the dark phase. Ageing astrocytes have been shown to undergo various morphological, genetic and functional changes that exacerbate neuroinflammation, impair glymphatic clearance of amyloid beta and increase blood–brain barrier permeability, thus making the brain more vulnerable to neurodegeneration and cognitive decline. There appear to be bi-directional relationships between increased sleep disturbance and neurodegeneration in older age that appear to be at least in part mediated by age-related changes in the brain’s astrocyte population.^85^

Microglia also contribute to neuroinflammation, including age-related exacerbation of neuroinflammation, neurodegeneration and sleep difficulties. Microglia are the innate immune cells of the central nervous system, which play important roles in maintaining brain homeostasis and modulating neuroinflammation. The fact that microglia morphology and activity changes with circadian rhythmicity suggests that these cells are likely also involved in regulating sleep.^86,87^ Microglia–neuron interactions are increasingly being recognized and characterized, with some of these interactions also impacting sleep and circadian functioning. For example, microglia prune neuronal synapses during sleep via purinergic (microglial P2Y12 receptor) signalling involving ATP by-products, thus supporting neuronal health and functioning.^87^ Age-related morphological and functional changes in microglia and an overall decline in the brain’s microglia population and phagocytic activity in older age contribute to circadian dysregulation and exacerbate neuroinflammation and neurodegeneration in a negative feedback loop.^86^ While neuroinflammation remains difficult to measure in living humans and current techniques cannot distinguish the contributions of individual cell populations, our study is the first to demonstrate relationships between MRSI markers and sleep outcomes in older adults that are consistent with emerging models of age-related increases in neuroinflammation and sleep problems.

In the ROI-based analyses, we did not observe the hypothesized correlations between temperature and metabolites in the dlPFC and executive functioning and processing speed, the hippocampus and memory, or between the dlPFC, hippocampus and thalamus and the sleep outcomes. The ROI-based results were largely consistent with the exploratory whole-brain analyses, which also did not detect associations specifically in these brain regions. However, higher total choline and mIns in brain regions adjacent to the dlPFC, specifically the superior and middle frontal gyri, were associated with slower processing speed and lower SE. The results reflect the increased sensitivity of exploratory analyses for detecting associations because they are not confined to predetermined ROI boundaries. The dlPFC is part of the middle frontal gyrus, so this region should remain an area of interest in further studies testing relationships between lifestyle variables and brain metabolites in healthy ageing.

The current study has important clinical implications. First, reliable biomarkers for neuroinflammation and neurodegeneration such as MRSI could be developed into diagnostic tools to identify individuals at risk of cognitive decline due to certain modifiable risk factors. Such a tool would allow for early intervention prior to the development of significant cognitive deficits. Here, we show associations between MRSI markers and lifestyle factors at the group level. Further studies with larger sample sizes are needed in this population to determine normative values so that MRSI could be used as a diagnostic tool in individual patients. Second, MRSI could be used to probe mechanisms underlying treatment effects, such as reduced neuroinflammation, remyelination or improved cellular metabolism. Studies aimed at improving sleep quality and physical fitness in older adults are effective at improving cognitive functioning,^88-90^ and MRSI could be used to identify and track the brain changes underlying these cognitive improvements.

### Limitations

The most important limitations of this study are its exploratory nature and modest sample size. Because few prior clinical studies have investigated neuroinflammation as a link between sleep and cognitive performance in older adults and its role in the development of cognitive impairment with age, the current study is intended to help establish MRSI as a potential non-invasive tool for moving this line of research forward in humans. Given the sample size along with the specific inclusion and exclusion criteria, the results of the current study may not be generalizable to the larger population of older adults, especially those with varying health conditions. The small sample size also reduces statistical power to detect real effects or, conversely, could result in spurious findings (false positives), and it did not allow us to statistically control for confounding variables such as level of education, diet and comorbid medical conditions, which could affect brain metabolite concentrations as well as cognitive performance. However, we note that participants were excluded if they reported major medical conditions that were not well controlled with medications. We also did not allow current smokers or users of illicit substances to participate, and all participants reported that their lifestyle included some level of physical activity. We have included a list of comorbid medical conditions, medications and other lifestyle variables in the supplementary materials.

Second, we did not include a control group. A comparative analysis with younger adults or individuals without cognitive concerns could offer more insights into age-related metabolic and cognitive changes and should be considered in future studies.

Third, because the cognitive assessments were administered in a separate visit than the PSG, we were not able to assess acute relationships between sleep and cognitive performance, and this could explain the unexpected absence of significant associations between sleep and cognition. Alternatively, limited variability in cognitive functioning in our sample could explain the lack of significant associations. Our inclusion criteria allowed for individuals with relatively impaired cognitive functioning (MoCA score of 18 or above) to participate in the study, but in practice, most of our participants were cognitively intact. SDs for the cognitive domain scores ranged from 0.52 (executive functioning) to 0.94 (visuospatial skills), indicating small variability in scores. In contrast, variability in sleep outcomes was much larger (SE: SD = 11.3; N3 time: SD = 31.3; N3%: SD = 7.8). We suggest that recruiting a sample with larger variability in cognitive abilities would increase our sensitivity to detect associations between sleep and cognition. Additionally, other variables related to sleep architecture, such as the amount of REM sleep and time spent in other non-REM sleep, could be assessed in future studies to gain a more complete understanding of the relationships between sleep and cognition in older adults.

Fourth, our recruitment strategy, which consisted of flyers, online advertisements and word-of-mouth, may introduce selection bias as only certain groups of people may have had access to this information. Future studies may consider using more systematic recruitment strategies to guard against this source of bias and ensure a representative sample.

Fifth, while PSG is the gold standard for quantifying objective sleep quality, people’s assessment of subjective sleep quality can differ from the PSG data. Collecting this information in the form of validated self-report measures would offer valuable insights and should be considered in future studies. Further, PSG was performed for only one night, which may not fully capture a participant’s typical sleep pattern in the home environment. However, we have previously shown in a patient population that a single night of laboratory-based PSG was significantly correlated with 14 days of actigraphy monitoring in the home environment, suggesting that PSG is a good approximation of a person’s typical sleep patterns.^91^

Finally, although the current study provides valuable insights into relationships between sleep architecture, brain metabolites and cognition in older adults, our cross-sectional design could not determine the directionality of these relationships.

## Conclusion

The current study assessed relationships between sleep, brain temperature and metabolites and cognitive performance in older adults. We report associations between tCho, mIns and NAA in widespread brain regions and cognitive functioning. We did not detect the expected associations between brain temperature and cognitive functioning or SE, suggesting that brain temperature may not be the best indicator of neuroinflammation, whereas brain metabolites appeared to be more sensitive. As expected, higher tCho and mIns in overlapping frontal regions were associated with lower SE, supporting the idea that abnormal metabolic and glial activity are risk factors for sleep disturbances in older adults. Overall, our results show that relationships between brain metabolites, sleep and cognition are complicated and warrant further characterization in larger, longitudinal studies.

## Supplementary Material

fcae245_Supplementary_Data

## Data Availability

Data will be made available upon reasonable request and Institutional Review Board approval. The code generated and used in this work is available in the supplementary materials.
