# Supplementary material for: Brain metabolites are associated with sleep architecture and cognitive functioning in older adults
Source: Brain Commun. 2024 Jul 19;6(4):fcae245. doi: 10.1093/braincomms/fcae245 (PMC11300014; doi:10.1093/braincomms/fcae245)
Supplement: fcae245_Supplementary_Data [file fcae245_supplementary_data.zip › 1_Supplementary Materials - Analysis Scripts.pdf]

```
### SETUP ENVIRONMENT ###

afni

FSLDIR=/usr/local/fsl
  . ${FSLDIR}/etc/fslconf/fsl.sh
  PATH=${FSLDIR}/bin:${PATH}
  export FSLDIR PATH
export FSLOUTPUTTYPE=NIFTI

### TEMP MAP SHIFTING, REALIGNING, SMOOTHING  ###

MYDIR=/Users/user/data

MYSUBJ=PID001
cd $MYDIR/$MYSUBJ
Myfirst=${MYSUBJ:0:1}
MyT1=$(find T1/$Myfirst*T1*acpc.nii)
Mytemp=$(find Temperature_files/$Myfirst*.nii)
\@Align_Centers -base $MyT1 -dset $Mytemp
rm $(find *.1D)
#mv $(find MRSt_files/*_shft.nii) Temperature_files
cd Temperature_files
for f in *_shft.nii ; do 3dcalc -a $f -expr '(a)*ispositive(a-
3)*isnegative(a-12)' -prefix T_$f; done
for f in T_*_shft.nii ; do 3dMedianFilter -irad 2 -prefix s2_$f $f; done

### CHO/CRE MAP SHIFTING, REALIGNING, SMOOTHING  ###
cd $MYDIR/$MYSUBJ
Myfirst=${MYSUBJ:0:1}
MyT1=$(find T1/$Myfirst*T1*acpc.nii)
MyCHO=$(find CHO_CRE_files/$Myfirst*.nii)
\@Align_Centers -base $MyT1 -dset $MyCHO
rm $(find *.1D)
cd CHO_CRE_files
for f in *_shft.nii ; do 3dMedianFilter -irad 2 -prefix smooth_$f $f;
done
for f in smooth_*_shft.nii ; do fslmaths $f -thr .00001 z$f; done

### MI/CRE MAP SHIFTING, REALIGNING, SMOOTHING  ###
cd $MYDIR/$MYSUBJ
Myfirst=${MYSUBJ:0:1}
MyT1=$(find T1/$Myfirst*T1*acpc.nii)
MyMINO=$(find MINO_CRE_files/$Myfirst*.nii)
\@Align_Centers -base $MyT1 -dset $MyMINO
rm $(find *.1D)
cd MINO_CRE_files
for f in *_shft.nii ; do 3dMedianFilter -irad 2 -prefix smooth_$f $f;
done
for f in smooth_*_shft.nii ; do fslmaths $f -thr .00001 z$f; done

### NAA/CRE MAP SHIFTING, REALIGNING, SMOOTHING  ###
cd $MYDIR/$MYSUBJ
Myfirst=${MYSUBJ:0:1}
```

```
MyT1=$(find T1/$Myfirst*T1*acpc.nii)
MyNAA=$(find NAA_CRE_files/$Myfirst*.nii)
\@Align_Centers -base $MyT1 -dset $MyNAA
rm $(find *.1D)
cd NAA_CRE_files
for f in *_shft.nii ; do 3dMedianFilter -irad 2 -prefix smooth_$f $f;
done
for f in smooth_*_shft.nii ; do fslmaths $f -thr .00001 z$f; done

### NEXT STEP: MATLAB SCRIPT TO RESIZE TEMP FILES!
```

```
function resize_img(imnames, Voxdim, BB, ismask)
%  resize_img -- resample images to have specified voxel dims and BBox
% resize_img(imnames, voxdim, bb, ismask)
%
% Output images will be prefixed with 'r', and will have voxel dimensions
% equal to voxdim. Use NaNs to determine voxdims from transformation
matrix
% of input image(s).
% If bb == nan(2,3), bounding box will include entire original image
% Origin will move appropriately. Use world_bb to compute bounding box
from
% a different image.
%
% Pass ismask=true to re-round binary mask values (avoid
% growing/shrinking masks due to linear interp)
%
% See also voxdim, world_bb

% Based on John Ashburner's reorient.m
% http://www.sph.umich.edu/~nichols/JohnsGems.html#Gem7
% http://www.sph.umich.edu/~nichols/JohnsGems5.html#Gem2
% Adapted by Ged Ridgway -- email bugs to drc.spm@gmail.com

% This version doesn't check spm_flip_analyze_images -- the handedness of
% the output image and matrix should match those of the input.

% Check spm version:
if exist('spm_select','file') % should be true for spm5
    spm5 = 1;
elseif exist('spm_get','file') % should be true for spm2
    spm5 = 0;
else
    error('Can''t find spm_get or spm_select; please add SPM to path')
end

spm_defaults;

% prompt for missing arguments
if ( ~exist('imnames','var') || isempty(char(imnames)) )
    if spm5
        imnames = spm_select(inf, 'image', 'Choose images to resize');
    else
        imnames = spm_get(inf, 'img', 'Choose images to resize');
    end
end
% check if inter fig already open, don't close later if so...
Fint = spm_figure('FindWin', 'Interactive'); Fnew = [];
if ( ~exist('Voxdim', 'var') || isempty(Voxdim) )
    Fnew = spm_figure('GetWin', 'Interactive');
    Voxdim = spm_input('Vox Dims (NaN for "as input")? ',...
        '+1', 'e', '[nan nan nan]', 3);
end
if ( ~exist('BB', 'var') || isempty(BB) )
    Fnew = spm_figure('GetWin', 'Interactive');
```

```
    BB = spm_input('Bound Box (NaN => original)? ',...
        '+1', 'e', '[nan nan nan; nan nan nan]', [2 3]);
end
if ~exist('ismask', 'var')
    ismask = false;
end
if isempty(ismask)
    ismask = false;
end

% reslice images one-by-one
vols = spm_vol(imnames);
for V=vols'
    % (copy to allow defaulting of NaNs differently for each volume)
    voxdim = Voxdim;
    bb = BB;
    % default voxdim to current volume's voxdim, (from mat parameters)
    if any(isnan(voxdim))
        vprm = spm_imatrix(V.mat);
        vvoxdim = vprm(7:9);
        voxdim(isnan(voxdim)) = vvoxdim(isnan(voxdim));
    end
    voxdim = voxdim(:)';

    mn = bb(1,:);
    mx = bb(2,:);
    % default BB to current volume's
    if any(isnan(bb(:)))
        vbb = world_bb(V);
        vmn = vbb(1,:);
        vmx = vbb(2,:);
        mn(isnan(mn)) = vmn(isnan(mn));
        mx(isnan(mx)) = vmx(isnan(mx));
    end

    % voxel [1 1 1] of output should map to BB mn
    % (the combination of matrices below first maps [1 1 1] to [0 0 0])
    mat = spm_matrix([mn 0 0 0 voxdim])*spm_matrix([-1 -1 -1]);
    % voxel-coords of BB mx gives number of voxels required
    % (round up if more than a tenth of a voxel over)
    imgdim = ceil(mat \ [mx 1]' - 0.1)';

    % output image
    VO            = V;
    [pth,nam,ext] = fileparts(V.fname);
    VO.fname      = fullfile(pth,['r' nam ext]);
    VO.dim(1:3)   = imgdim(1:3);
    VO.mat        = mat;
    VO = spm_create_vol(VO);
    spm_progress_bar('Init',imgdim(3),'reslicing...','planes completed');
    for i = 1:imgdim(3)
        M = inv(spm_matrix([0 0 -i])*inv(VO.mat)*V.mat);
        img = spm_slice_vol(V, M, imgdim(1:2), 1); % (linear interp)
        if ismask
```

```
            img = round(img);
        end
        spm_write_plane(VO, img, i);
        spm_progress_bar('Set', i)
    end
    spm_progress_bar('Clear');
end
% call spm_close_vol if spm2
if ~spm5
    spm_close_vol(VO);
end
if (isempty(Fint) && ~isempty(Fnew))
    % interactive figure was opened by this script, so close it again.
    close(Fnew);
end
disp('Done.')


%%%%%%%%%%%%%%%%%%%%%%%%%%%%%%%%%%%%%%%%%%%%%%%%%%%%%%%%%%%%%%%%%%%%%%%%%
%
function bb = world_bb(V)
%  world-bb -- get bounding box in world (mm) coordinates

d = V.dim(1:3);
% corners in voxel-space
c = [ 1    1    1    1
    1    1    d(3) 1
    1    d(2) 1    1
    1    d(2) d(3) 1
    d(1) 1    1    1
    d(1) 1    d(3) 1
    d(1) d(2) 1    1
    d(1) d(2) d(3) 1 ]';
% corners in world-space
tc = V.mat(1:3,1:4)*c;

% bounding box (world) min and max
mn = min(tc,[],2)';
mx = max(tc,[],2)';
bb = [mn; mx];
```

```
restoredefaultpath
clearvars
clc
addpath(genpath('/Users/user/Documents/Realign_resize_TmapCREscripts/'));
addpath(genpath('/Users/user/Documents/MATLAB/spm12/'));
rmpath(genpath('/Users/user/Documents/MATLAB/spm12/external/fieldtrip/com
pat/'));

% nproc=feature('numcores');
% parpool('local',nproc);

spm('defaults', 'fmri'); % Initiate SPM
spm_jobman('initcfg');


data_path = '/Users/user/data';

list_subj = dir(strcat(data_path,'/*/Temperature_files/s2*Cre*.nii'));
%list_subj = dir(strcat(data_path,'/*/CHO_CRE_files/zsmooth*.nii'));
%list_subj = dir(strcat(data_path,'*/MINO_CRE_files/zsmooth*.nii'));

for ii=1:numel(list_subj)

    mymask = strcat(list_subj(ii).folder,filesep,list_subj(ii).name);

    imnames = mymask;
    Voxdim = [2 2 2];
    BB =  [-90, -126, -72; 90, 90, 108];% [-78 -112 -70; 78 76 85] for BB
(bounding box)
    resize_img(imnames,Voxdim,BB);

end
```

```
#!/bin/bash -f

basedir=/path/to/data

3dttest++ \
-prefix MRSt_McKnight_Tmap_regression_attention_domain \
-mask ${basedir}/group_stats/brain_mask.nii \
-resid residuals \
-setA HBC \
HBC01
${basedir}/HBC01/Temperature_files/s8_rs2_T_HBC01_Tmap_Cre_Norm_shft.nii
\
HBC02
${basedir}/HBC02/Temperature_files/s8_rs2_T_HBC02_Tmap_Cre_Norm_shft.nii
\
HBC03
${basedir}/HBC03/Temperature_files/s8_rs2_T_HBC03_Tmap_Cre_Norm_shft.nii
\
HBC05
${basedir}/HBC05/Temperature_files/s8_rs2_T_HBC05_Tmap_Cre_Norm_shft.nii
\
HBC06
${basedir}/HBC06/Temperature_files/s8_rs2_T_HBC06_Tmap_Cre_Norm_shft.nii
\
HBC07
${basedir}/HBC07/Temperature_files/s8_rs2_T_HBC07_Tmap_Cre_Norm_shft.nii
\
HBC08
${basedir}/HBC08/Temperature_files/s8_rs2_T_HBC08_Tmap_Cre_Norm_shft.nii
\
HBC09
${basedir}/HBC09/Temperature_files/s8_rs2_T_HBC09_Tmap_Cre_Norm_shft.nii
\
HBC10
${basedir}/HBC10/Temperature_files/s8_rs2_T_HBC10_Tmap_Cre_Norm_shft.nii
\
HBC12
${basedir}/HBC12/Temperature_files/s8_rs2_T_HBC12_Tmap_Cre_Norm_shft.nii
\
HBC14
${basedir}/HBC14/Temperature_files/s8_rs2_T_HBC14_Tmap_Cre_Norm_shft.nii
\
HBC15
${basedir}/HBC15/Temperature_files/s8_rs2_T_HBC15_Tmap_Cre_Norm_shft.nii
\
HBC16
${basedir}/HBC16/Temperature_files/s8_rs2_T_HBC16_Tmap_Cre_Norm_shft.nii
\
HBC17
${basedir}/HBC17/Temperature_files/s8_rs2_T_HBC17_Tmap_Cre_Norm_shft.nii
\
HBC18
${basedir}/HBC18/Temperature_files/s8_rs2_T_HBC18_Tmap_Cre_Norm_shft.nii
\
```

```
HBC20
${basedir}/HBC20/Temperature_files/s8_rs2_T_HBC20_Tmap_Cre_Norm_shft.nii
\
HBC21
${basedir}/HBC21/Temperature_files/s8_rs2_T_HBC21_Tmap_Cre_Norm_shft.nii
\
HBC22
${basedir}/HBC22/Temperature_files/s8_rs2_T_HBC22_Tmap_Cre_Norm_shft.nii
\
HBC23
${basedir}/HBC23/Temperature_files/s8_rs2_T_HBC23_Tmap_Cre_Norm_shft.nii
\
HBC24
${basedir}/HBC24/Temperature_files/s8_rs2_T_HBC24_Tmap_Cre_Norm_shft.nii
\
HC01
${basedir}/HC01/Temperature_files/s8_rs2_T_HC01_Tmap_Cre_Norm_shft.nii \
HC02
${basedir}/HC02/Temperature_files/s8_rs2_T_HC02_Tmap_Cre_Norm_shft.nii \
-covariates covariates.txt'[0,2,3,10]'
```

```
#!/bin/bash

3dFWHMx \
-mask /path/to/data/group_stats/brain_mask.nii \
-input residuals+tlrc \
>> 3dFWHMx_results_brain_mask.txt

rm 3dFWHMx.1D*
```

```
## 3dClustSim using brain mask
3dClustSim \
 -mask /path/to/data/group_stats/brain_mask.nii \
 -acf 0.631911  12.5706  21.2631 \
-athr 0.05 -nodec \
 -prefix ClustSim_results_brain_mask
```

```
#!/bin/bash

## Base variables. Change if needed
subject=$1
tempdir=/path/to/data/group_stats/whole-brain_analyses/
outdir=${tempdir}/cluster_metrics
datadir=/path/to/data

#CHO_CRE
#3dROIstats -mask
${tempdir}/CHO_regression_attention_domain/CHO_attn_wm_Clust_mask+tlrc
${datadir}/${subject}/CHO_CRE_files/s8_rzsmooth_${subject}_CHO_CR_Norm_sh
ft.nii > ${outdir}/CHO_attention_wm/${subject}_clust_attn_wm_cho.txt
#3dROIstats -mask
${tempdir}/CHO_regression_language_domain/CHO_language_Clust_mask+tlrc
${datadir}/${subject}/CHO_CRE_files/s8_rzsmooth_${subject}_CHO_CR_Norm_sh
ft.nii > ${outdir}/CHO_language/${subject}_clust_language_cho.txt
#3dROIstats -mask
${tempdir}/CHO_regression_proc_speed_domain/CHO_proc_speed_Clust_mask+tlr
c
${datadir}/${subject}/CHO_CRE_files/s8_rzsmooth_${subject}_CHO_CR_Norm_sh
ft.nii > ${outdir}/CHO_proc_speed/${subject}_clust_proc_speed_cho.txt
3dROIstats -mask ${tempdir}/CHO_regression_SE/CHO_SE_Clust_mask+tlrc
${datadir}/${subject}/CHO_CRE_files/s8_rzsmooth_${subject}_CHO_CR_Norm_sh
ft.nii > ${outdir}/CHO_SE/${subject}_clust_se_cho.txt

#MINO_CRE
#3dROIstats -mask
${tempdir}/MINO_regression_language_domain/MINO_language_Clust_mask+tlrc
${datadir}/${subject}/MINO_CRE_files/s8_rzsmooth_${subject}_MINO_CR_Norm_
shft.nii > ${outdir}/MINO_language/${subject}_clust_language_mino.txt
#3dROIstats -mask
${tempdir}/MINO_regression_proc_speed_domain/MINO_proc_speed_Clust_mask+t
lrc
${datadir}/${subject}/MINO_CRE_files/s8_rzsmooth_${subject}_MINO_CR_Norm_
shft.nii > ${outdir}/MINO_proc_speed/${subject}_clust_proc_speed_mino.txt
3dROIstats -mask ${tempdir}/MINO_regression_SE/MINO_SE_Clust_mask+tlrc
${datadir}/${subject}/MINO_CRE_files/s8_rzsmooth_${subject}_MINO_CR_Norm_
shft.nii > ${outdir}/MINO_SE/${subject}_clust_se_mino.txt

#NAA
#3dROIstats -mask
${tempdir}/NAA_regression_vis_spatial_domain/NAA_vis_spatial_Clust_mask+t
lrc ${datadir}/${subject}/NAA/s8_rzsmooth_${subject}_NAA_CR_Norm_shft.nii
> ${outdir}/NAA_vis_spatial/${subject}_clust_vis_spatial_naa.txt
```
