# Supplementary figures and images for: Brain metabolites are associated with sleep architecture and cognitive functioning in older adults
Source: Brain Commun. 2024 Jul 19;6(4):fcae245. doi: 10.1093/braincomms/fcae245 (PMC11300014; doi:10.1093/braincomms/fcae245)

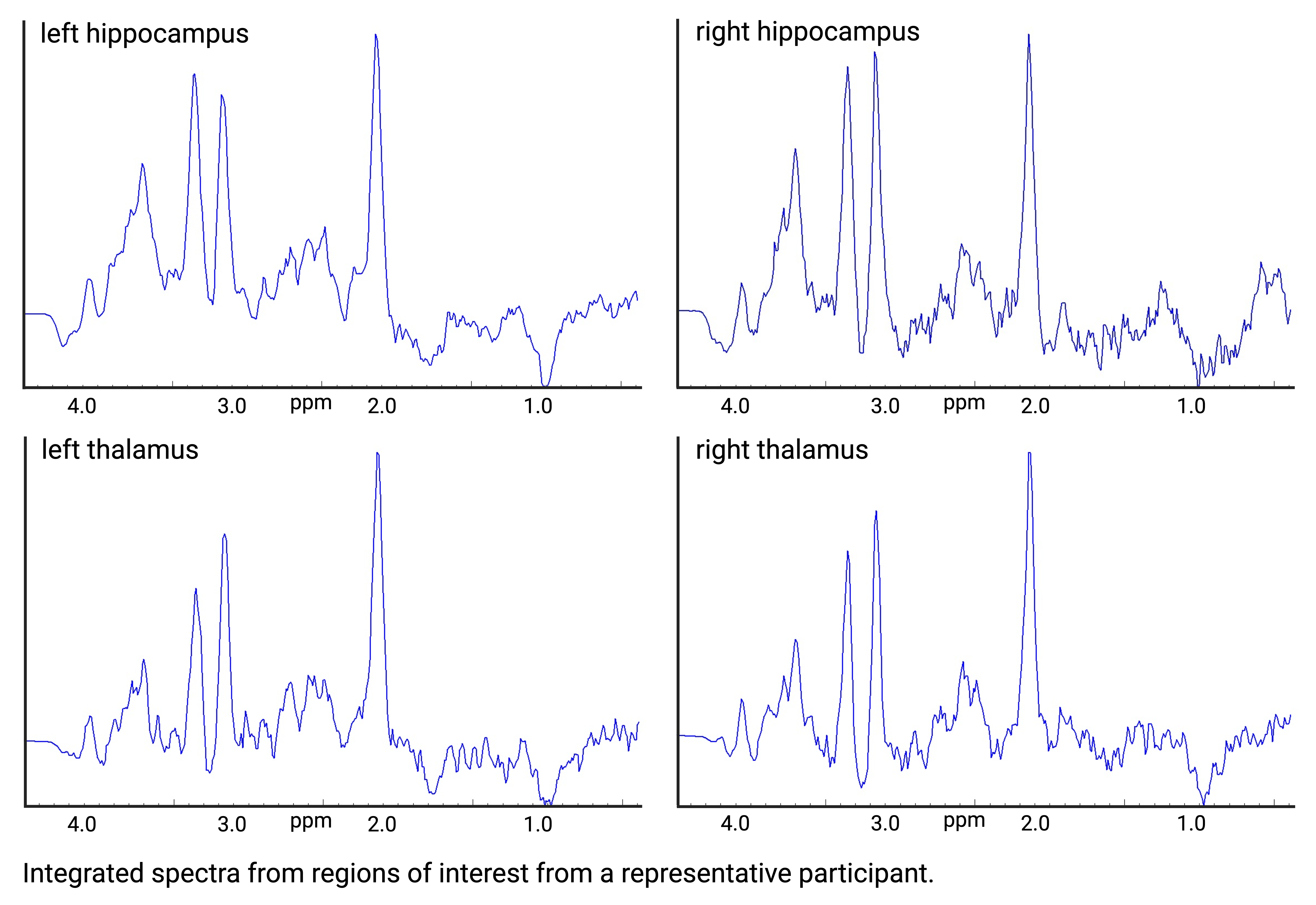

Supplement: fcae245_Supplementary_Data [file fcae245_supplementary_data.zip › 4_Supplementary Figure 2 - MRS Spectrum.png]

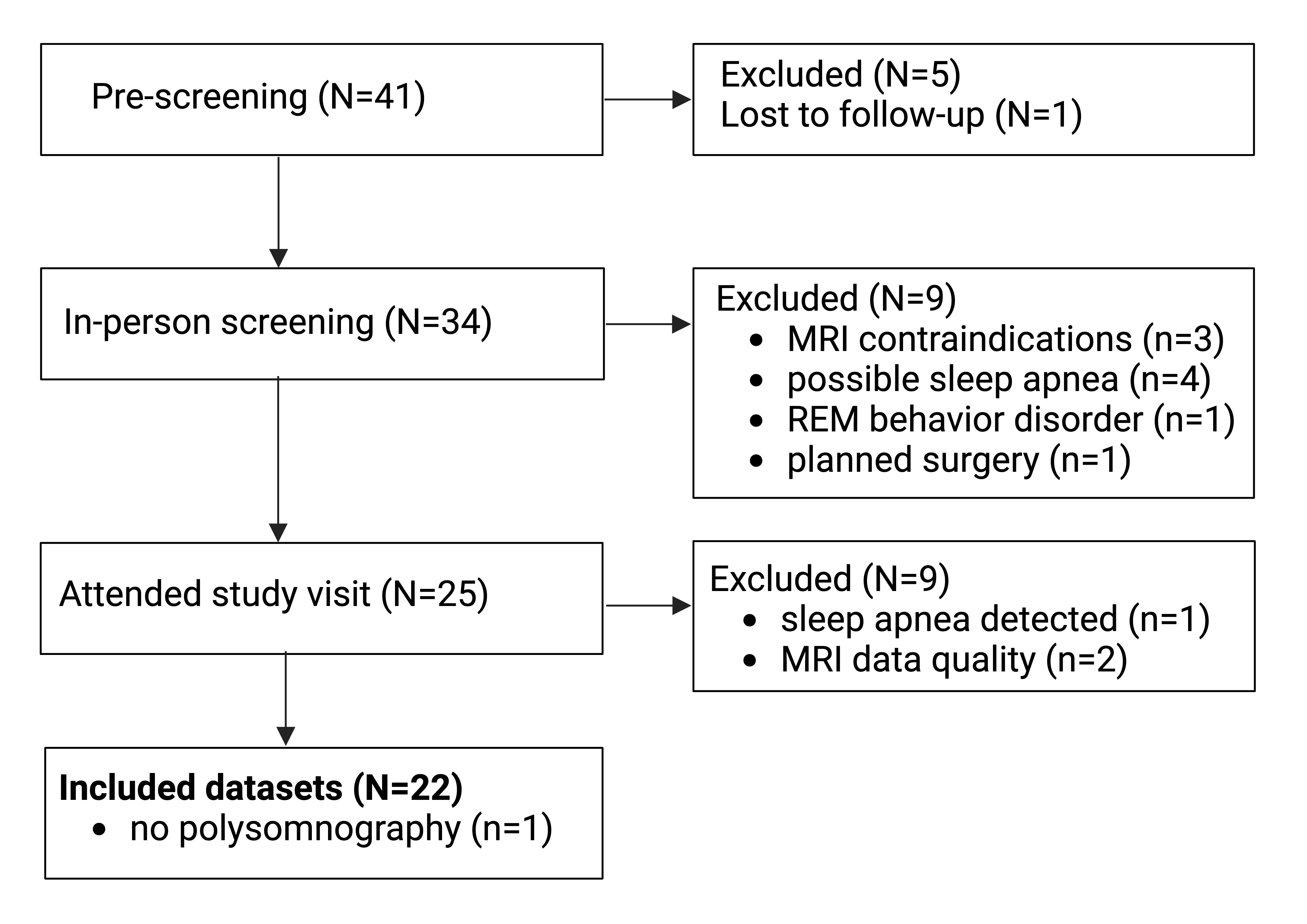

Supplement: fcae245_Supplementary_Data [file fcae245_supplementary_data.zip › 2_Supplementary Figure 1 - Flow Chart.png]
